# Baseline indices of cardiorespiratory fitness for predicting acute mountain sickness: an interpretable four-task ensemble model

**DOI:** 10.3389/fpubh.2026.1883321

**Published:** 2026-09-22

**Authors:** Shuai Wang, Fengbo Miao, Chengli Tao, Yupeng Liu, Qi Mao, Caitong Zhao, Hualin Zhang, Renzheng Chen, Ning Li

**Affiliations:** 1 Department of Oral and Maxillofacial Surgery, the General Hospital of Northern Theater Command, Shenyang, China; 2 School of Electronics and Information Engineering, Tiangong University, Tianjin, China; 3 Department of Cardiology, the 967th Hospital of Joint Logistics Support Force of Chinese PLA, Dalian, China; 4 Department of Critical Care Medicine, the 967th Hospital of Joint Logistics Support Force of Chinese PLA, Dalian, China; 5 Department of Emergency, the 967th Hospital of Joint Logistics Support Force of Chinese PLA, Dalian, China; 6 Department of Quality Control, The General Hospital of Northern Theater Command, Shenyang, China; 7 Department of Infection Control, The 967th Hospital of Joint Logistics Support Force of Chinese PLA, Dalian, China; 8 Department of Cardiac Surgery, The General Hospital of Northern Theater Command, Shenyang, China

**Keywords:** acute mountain sickness, cardiorespiratory fitness, high-altitude acclimatization, machine learning, prediction model, SHapley additive exPlanation

## Abstract

**Background:**

Acute mountain sickness (AMS) poses formidable challenges for individuals venturing into high-altitude (HA) environments. Early and precise prediction of AMS is crucial for the timely identification of high-risk individuals and the development of effective treatment strategies. We sought to develop and validate an interpretable Acute Mountain Sickness Four-Task Ensemble (AMS-FTE) model for predicting AMS occurrence and severity according to the old and new LLS definitions.

**Methods:**

This study enrolled 330 healthy subjects who underwent rapid exposure to HA. Questionnaire surveys utilizing the Lake Louise Score for AMS were administered upon arrival. Five machine learning (ML) algorithms were evaluated independently for each prediction task, after which RF, AdaBoost, and LightGBM were combined to construct the AMS-FTE framework. To enhance interpretability, the SHapley Additive exPlanation method illuminated feature importance. The area under the receiver operating characteristic curve and decision curve analysis were evaluated to calculate predictive power and assess the model’s clinical applicability.

**Results:**

Following algorithm comparison, AMS-FTE model was constructed to provide unified predictions for the four AMS-related tasks. Based on feature importance rankings, interpretable final model was developed for each task using the top 5 contributing features, which were associated with baseline cardiopulmonary fitness (post-exercise heart rate, post-exercise SpO_2_, AT-RCP time, peak VO_2_, and ΔVO_2_ AT-RCP). This model demonstrated robust predictive accuracy for AMS, and showed significant clinical net benefit probability ranges, demonstrating strong clinical applicability.

**Conclusion:**

The predictive value of baseline cardiorespiratory fitness parameters proved valuable in distinguishing AMS and non-AMS subjects, as revealed through an interpretable ML model and a support system. This will enable earlier diagnosis for AMS, and more targeted interventions for acute altitude illness.

## Graphical abstract

**Figure:**
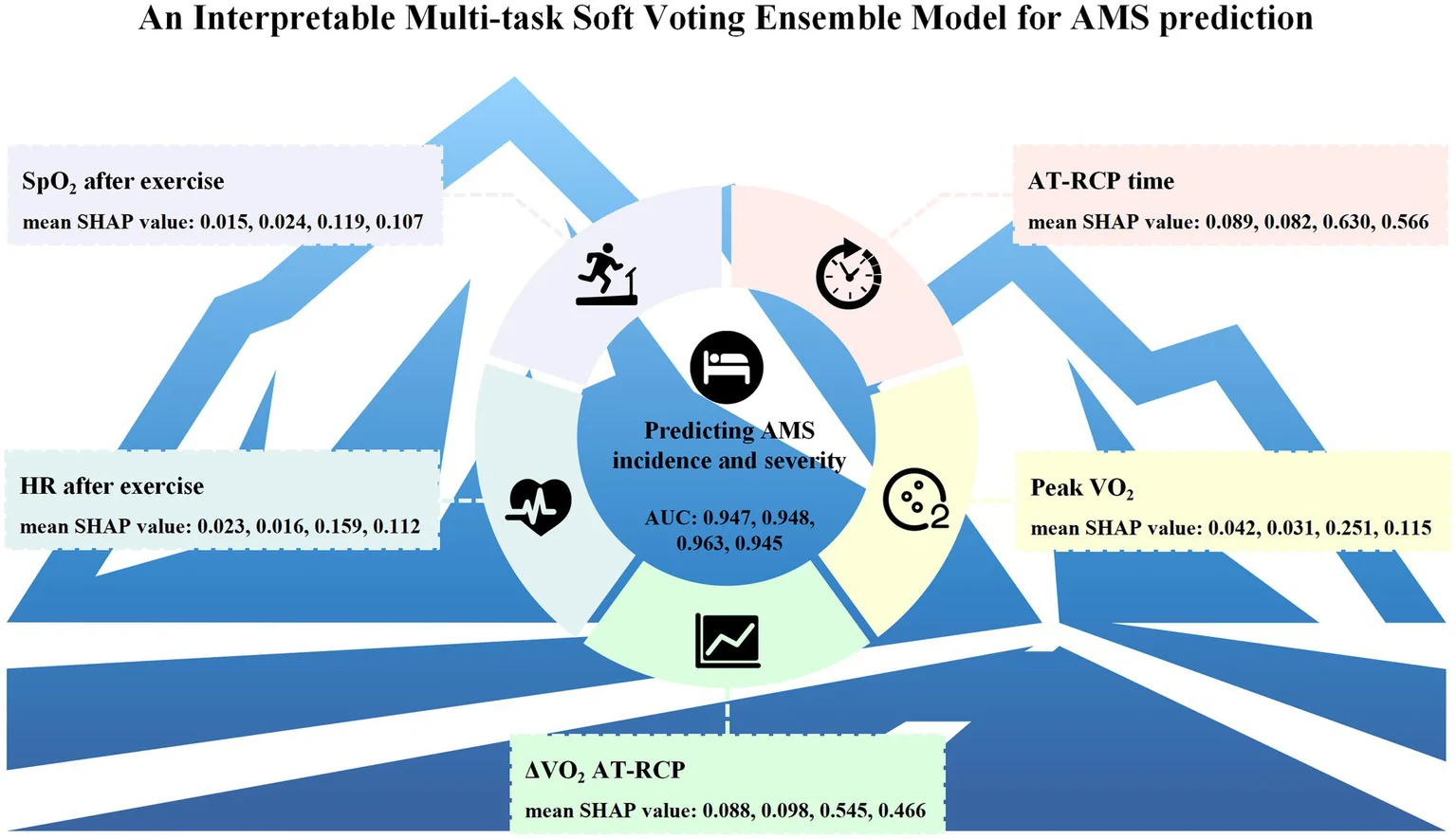

## Introduction

Hypobaric hypoxia presents a substantial challenge at high altitudes (HA). The cardiovascular and respiratory systems adapt to hypoxic stress through intrinsic self-regulation, with hyperventilation and an elevated heart rate (HR) emerging as the most prominent physiological changes under these conditions (1–3). Maladaptation can trigger acute mountain sickness (AMS), which is believed to be linked to abnormal cardiopulmonary function (4–6). The prevalence of AMS may soar dramatically, reaching a staggering 40–90% among unacclimatized individuals who ascend rapidly to altitudes of 4,500-6000 m (7, 8). Post-exercise physiological indicators, which to some extent reflect cardiopulmonary fitness, have recently been demonstrated to correlate closely with the onset and progression of AMS (9). Moreover, Cardiopulmonary exercise testing (CPET), the precise assessment of cardiorespiratory fitness, serves as an accurate and effective method for evaluating compensatory responses during acute HA exposure (9, 10).

During CPET, the ventilatory profile is characterized by 2 distinct inflection points: the anaerobic threshold (AT) and the respiratory compensation point (RCP) (11). The interval between AT and RCP (AT-RCP time) represents the isocapnic buffering phase, during which rising lactate concentrations are buffered by bicarbonate to prevent acidosis (12). AT-RCP time and the oxygen uptake between AT and RCP (∆VO_2_ AT-RCP) demonstrate the capacity for continued exertion before breathlessness emerges post-AT (13). Most CPET studies confirm that acute hypobaric hypoxia exposure diminishes cardiorespiratory fitness in HA environments, reducing peak VO_2_, VO_2_ at AT and RCP, and the VO_2_/work rate (WR) slope (14, 15). However, whether these parameters can effectively predict AMS remains unclear.

Machine learning (ML) focuses on constructing application programs. It employs systematic algorithms and statistical processing steps to train models with vast amounts of data, enabling them to identify patterns and features (16, 17). This capability, in turn, allows for predictions to be made with new data. However, many prediction models grapple with interpretability issues, creating challenging “black box” dilemmas that restrict their real-world utility (18). The interpretability of ML models holds paramount importance in clinical decision-making, where understanding the underlying rationale for predictions is just as vital as achieving high predictive accuracy. To bridge this critical gap, the SHapley Additive exPlanations (SHAP) method has gained considerable traction as a powerful tool for enhancing model transparency and has firmly established itself as a cornerstone methodology for developing trustworthy, interpretable ML models within clinical research (19, 20).

Therefore, in this study, we sought to develop an interpretable, Acute Mountain Sickness Four-Task Ensemble (AMS-FTE) prediction model. By integrating both the new and old versions of the Lake Louise Score (LLS) to assess incidence and severity, achieved more comprehensive disease prediction.

## Methods

### Study populations and protocols

This retrospective study comprised 330 normal adult males who had predominantly resided in lowland areas (LA). All participants were subjected to an acute exposure to HA conditions in Lhasa, Tibet, China, at an elevation of 3,700 m, from LA in Chengdu, Sichuan, China, at 500 m, via air travel within a span of 3 h, distributed across various batches over 1 week. Prior to their expedition, they underwent a thorough medical evaluation, and subjects with a history of chronic diseases (e.g., cardiopulmonary diseases), those who have been exposed to HA within the last 6 months, or those currently taking medications that may impair cardiopulmonary function were excluded. All subjects underwent baseline physiological assessments, including ambulatory blood pressure (ABP) monitoring, echocardiography, electrocardiography (ECG), and CPET, at LA. All baseline measurements were completed within 1 week prior to HA exposure, with all measurements scheduled uniformly between 8:00 and 10:00 a.m. They were instructed to refrain from engaging in unusual physical activities and to follow a standard schedule. Additionally, they were required to abstain from smoking and alcohol consumption, with medication permitted only for special treatment needs.

#### Echocardiography examination

Echocardiographic examination was conducted using an ultrasound machine (CX50, Philips Ultrasound System, Andover, MA, United States). Images were digitally saved for subsequent offline analysis with software (QLAB 10.5, Philips Healthcare, Andover, MA, United States). Ejection fraction (EF) was calculated from the LV volume data. The mitral inflow pattern at the tips level was analyzed for peak early diastolic velocity (E) and late diastolic velocity (A), as well as the E/A ratio. The mitral annulus early diastolic velocity (e’) was measured at both the septal and lateral mitral annulus, and the E/e’ ratio was calculated using the mean septal and lateral E/e’ ratio. The global longitudinal strain (GLS) was determined by averaging all regional peak longitudinal strain values obtained from 2-chamber, 3-chamber, and 4-chamber apical views. The global circumferential strain (GCS) was evaluated as the average of 3 regional values. Measurements were averaged over three cardiac cycles.

#### ECG examination

Standard 12-lead ECGs (custo-Cardio 3000BT-A, Cortex, Ottobrunn, Germany) were recorded at a speed of 25 mm/s and with an amplitude of 10 mm/mV. The software (Custo Diagnostic 5.5.9 Build 214, Custo Med GmbH, Ottobrunn, Germany) automatically obtained the average resting voltages and durations of the segments. Two trained physicians manually analyzed the same HR over a 20-s period in standard and precordial leads on a high-resolution computer screen. Average values were obtained when there was a discrepancy between measurements. The QT interval was measured from the onset of the QRS complex to the end of the T-wave, and the TpTe interval was defined as the interval between the peak of the T-wave and the end of the T-wave. The Bazett formula was used to correct the QT interval (QTc) and TpTe interval (TpTec). When the peak of the T-wave was not clearly definable, that is, when the T-wave was either flattened or had a notch, the midpoint of the flattened top or the nadir of the notch was taken as the T-wave value. The end of the T-wave was determined using the tail method.

### CPET

CPET was performed using a stationary bicycle (EC3000e, Customed, Bitz, Germany) and a breath-by-breath gas analyzer (Metalyzer 3B, Cortex, Ottobrunn, Germany). Subjects were instructed to avoid strenuous exercise for 24 h, refrain from consuming caffeine-containing beverages, abstain from smoking and alcohol consumption, and ensure adequate sleep before the tests. The cycle ergometry exercise protocol was characterized by at least 3 min of rest, 3 min of unloaded exercise, a continual increase in resistance at 25 W/min, and 3 min of unloaded cycling. Data output directly from the system (MetaSoft® Studio, Cortex Biophysik GmbH, Ottobrunn, Germany) were processed in Excel spreadsheets in the appropriate format by a physician and a skilled technician, which facilitated subsequent analysis. Termination criteria of CPET were: 1. Symptoms or signs of impaired breathing and circulatory function (pale face, clammy skin, cyanosis, dizziness, chest pain, nausea, severe dyspnea, precordial pain with ischemic ST-segment changes greater than 2 mm, severe hypertension (240/140 mmHg), systolic blood pressure (SBP) drop >10 mmHg; 2. Occurrence of severe fatigue or severe leg pain, preventing the participants from pedaling. The AT was determined through V-slope analysis of increases in VO_2_ and VCO_2_, which was further confirmed by the distinctive changes observed in the ventilatory equivalent and O_2_ end-tidal pressure. Similarly, the RCP was identified when there was an increase in ventilatory equivalent/VCO_2_, along with a corresponding decrease in CO_2_ end-tidal pressure. In addition, hemodynamic and oxygenation parameters, including blood pressure (BP), HR and percutaneous oxygen saturation (SpO_2_), were measured immediately following the termination of unloaded exercise (during the first minute after the unloaded cycling recovery phase) to assess acute recovery kinetics.

### LLS

After arriving at HA, within 12–24 h, both the original 1993 (old) and revised 2018 (new) versions of the LLS were employed to evaluate the presence and severity of AMS. Symptoms were each assigned score of 0 (none), 1 (mild), 2 (moderate), or 3 (severe). A total score of ≥ 3, alongside headache, constituted a diagnosis of AMS (21).

### Model development and feature selection

Prior to model training, all continuous features were assessed for missing values and outliers using the IQR. Continuous predictors were standardized using Z-score normalization within the modeling pipeline. For each of the four prediction tasks (AMS diagnosed by the old LLS, AMS diagnosed by the new LLS, AMS score using the old LLS, and AMS score using the new LLS), the 330 participants were first divided at the participant level into a training set and a held-out test set at an 80:20 ratio using stratified random sampling. Stratification was based on the corresponding binary AMS outcome defined by the corresponding LLS version. Five-fold stratified cross-validation was subsequently performed exclusively within the 80% training set, with four folds used for model fitting and one fold used for internal validation in each iteration. The held-out 20% test set was then used for performance evaluation. All preprocessing steps were applied consistently across the 80:20 stratified split, and were fitted only on the training set to prevent data leakage. Thirty-three features (subject-to-feature ratio of 10:1) formed the foundation for building model. We evaluated 5 ML algorithms: support vector machine (SVM), random forest (RF), light gradient boosting machine (LightGBM), adaptive boosting (AdaBoost), and extreme gradient boosting (XGBoost). All models were implemented using fixed hyperparameter settings, with a random seed of 42 where applicable. RF used 120 trees, a maximum depth of 6, and a minimum of 2 samples per leaf. AdaBoost used 120 estimators and a learning rate of 0.05. LightGBM used 120 estimators, a maximum depth of 4, a learning rate of 0.05, and subsampling and feature-sampling ratios of 0.85. XGBoost used 120 estimators, a maximum depth of 3, a learning rate of 0.05, and subsampling and feature-sampling ratios of 0.85. The SVM used a radial basis function kernel, C = 1.0, and the scale-based gamma setting. The proportions of AMS cases were 38.2 and 34.2% according to the two versions of LLS respectively, indicating moderate rather than severe class imbalance. No synthetic oversampling, undersampling, or class-weight adjustment was applied because these approaches may introduce additional assumptions or artificial samples in a relatively small clinical cohort. Model performance was evaluated using accuracy, precision, recall, and F1-score for classification tasks; mean absolute error (MAE), root mean square error (RMSE) and R^2^ for regression tasks. Following the comparison of the five candidate algorithms, RF, AdaBoost, and LightGBM were retained as the three constituent algorithms of the final ensemble framework. Independent copies of the three constituent algorithms were fitted separately for the four prediction tasks. For classification task (k), the positive-class probabilities generated by the three constituent classifiers were combined using equal-weight soft voting:

$${\hat{y}}_{k}(x)=\frac{{\hat{y}}_{\mathrm{RF},k}(x)+{\hat{y}}_{\text{AdaBoost},k}(x)+{\hat{y}}_{\text{LightGBM},k}(x)}{3}$$

For regression task (k), the three continuous predictions were combined using equal-weight arithmetic averaging:

$${\hat{p}}_{k}(x)=\frac{{\hat{p}}_{\mathrm{RF},k}(x)+{\hat{p}}_{\text{AdaBoost},k}(x)+{\hat{p}}_{\text{LightGBM},k}(x)}{3}$$

Within each task-specific ensemble, the predictions generated by RF, AdaBoost, and LightGBM were combined using equal aggregation weights. Class-probability averaging was used for the classification tasks, whereas arithmetic averaging of continuous outputs was used for the regression tasks. Each base learner was fitted independently using its algorithm-specific training criterion or objective. Specifically, RF used Gini impurity as the node-splitting criterion for classification and squared error for regression; AdaBoost used its standard binary adaptive-boosting formulation for classification and linear loss for regression; and LightGBM optimized binary log loss for classification and L2 loss for regression. Because the four outcome-specific ensembles were developed independently, the AMS-FTE model did not define or optimize a joint cross-task objective, and no fitted parameters, tree structures, residuals, gradients, or other training information were shared or transferred across tasks. Instead, the four modeling pipelines followed a common specification. At deployment, the four independently fitted ensembles were integrated through a unified inference interface to generate the four prediction outputs. This software-level integration did not introduce cross-task parameter sharing or joint optimization. Global SHAP importance, calculated from the mean absolute SHA*p* values, was used to quantify and rank the relative contribution of each variable to the ensemble predictions. Feature selection was conducted by combining clinical expert judgment with model-based validation. This process aimed not only to identify the most discriminative variables but also to eliminate redundant features that could introduce variance and compromise model generalizability, and the predictive performance was evaluated using the area under the receiver operating characteristic curve (AUC). SHAP analysis and feature-number comparison were performed only within the training set before final evaluation on the held-out test set.

### Statistical analysis

Statistical analyses were performed using Python (v3.11.3; Python Software Foundation) and R (v4.3.0; R Foundation for Statistical Computing). Continuous variables are presented as mean ± standard deviation (SD) or median [interquartile range (IQR)], while categorical variables are expressed as frequencies (%). Differences between groups for normally distributed continuous data were evaluated using independent-sample t-tests; conversely, the Mann–Whitney U test was employed for non-normally distributed data. Categorical data comparisons utilized the chi-square test, with continuity correction or Fisher’s exact test applied where appropriate. Decision curve analysis (DCA) was conducted using predictions generated by the four task-specific ensembles within the final five-feature AMS-FTE framework. For each participant, the out-of-fold prediction was generated by a model trained on the other four folds, ensuring that the participant’s data were not used for model fitting. Positive-class probabilities were used for the two classification tasks. For the two regression tasks, the out-of-fold predicted AMS scores were linearly rescaled to a 0–1 risk scale using the observed score range derived exclusively from the corresponding training fold and were evaluated against the matching binary AMS outcome. The transformed values were treated as model-derived normalized risk scores for decision analysis. At decision threshold pt., net benefit (NB) was calculated as:

$$\mathrm{NB}(p_{t})=\frac{\mathrm{TP}}{N}-\frac{\mathrm{FP}}{N}\times \frac{p_{t}}{1-p_{t}}$$

Where TP and FP denote the numbers of true-positive and false-positive decisions, respectively, and N denotes the total number of participants. The net benefit of the treat-none strategy was defined as 0, whereas that of the treat-all strategy was calculated as:

$$NB_{\mathrm{all}}(p_{t})=\text{prevalence}-(1-\text{prevalence})\times \frac{p_{t}}{1-p_{t}}$$

Where prevalence denotes the prevalence of AMS according to the corresponding LLS definition. For the classification tasks, pt. represented the predicted-probability threshold; for the regression-derived analyses, it represented the corresponding threshold on the normalized risk scale. Bonferroni correction was applied for multiple pairwise comparisons, and a two-sided *p* < 0.05 was considered statistically significant.

## Results

### Basic characteristics

Upon reaching altitude, the old LLS criteria diagnosed 126 subjects (38.2%) with AMS, compared to 113 subjects (34.2%) identified using the new LLS. There were no statistically significant differences in demographic characteristics between the AMS group and the non-AMS group. Post-exercise HR [96.00 (87.00, 104.00) vs. 88.50 (85.00, 93.00), *p* < 0.001] and the VO_2_/WR slope [9.82 (9.16, 10.27) vs. 9.20 (8.50, 9.72), *p* < 0.001) were substantially higher in the AMS group diagnosed by the old LLS relative to the non-AMS group. Conversely, post-exercise SpO_2_ [93.00 (92.00, 95.00) vs. 95.00 (94.00, 97.00), *p* < 0.001], AT-RCP time [157.10 (147.41, 168.06) vs. 175.91 (168.12, 186.70), *p* < 0.001], peak VO_2_ (31.27 ± 2.48 vs. 34.06 ± 2.70, *p* < 0.001), and ΔVO_2_ AT-RCP [9.90 (8.45, 11.21) vs. 12.38 (11.09, 13.91), *p* < 0.001] were notably lower among subjects diagnosed with AMS by the old LLS criteria versus those without AMS. These distinct patterns were similarly observed and maintained in the groups diagnosed using the new LLS criteria for AMS. Notably, no statistically significant differences were observed in resting SpO_2_, HR or resting blood pressure between groups, suggesting that dynamic stress-testing is essential to unmask latent cardiopulmonary vulnerabilities predisposing to AMS (Table 1).

**Table 1:** Demographic characteristics and physiological data among the individuals with and without AMS.

Variables	Total (*n* = 330)	Old LLS		*p* value	New LLS		*p* value
		AMS (*n* = 126)	Non-AMS (*n* = 204)		AMS (*n* = 113)	Non-AMS (*n* = 217)	
AMS score of old LLS	2.00 [1.00, 4.00]	4.00 [4.00, 5.00]	1.00 [0.00, 2.00]	<0.001	–	–	–
AMS score of new LLS	2.00 [1.00, 3.00]	–	–	–	4.00 [3.00, 4.00]	1.00 [0.00, 2.00]	<0.001
Age, year	20.00 [19.00, 23.00]	21.00 [20.00, 23.00]	20.00 [19.00, 22.00]	0.063	21.00 [20.00, 23.00]	20.00 [19.00, 22.00]	0.054
BMI, kg/m^2^	21.22 [19.85, 22.49]	21.39 [20.16, 22.45]	20.99 [19.72, 22.49]	0.296	21.45 [20.20, 22.31]	20.99 [19.72, 22.49]	0.202
Males	330 (100.0%)	126 (100.0%)	204 (100.0%)	1.000	113 (100.0%)	217 (100.0%)	1.000
Tibetan	5 (1.5%)	2 (1.6%)	3 (1.5%)	1.000	1 (0.9%)	4 (1.8%)	0.840
History of drinking	236 (71.5%)	88 (69.8%)	148 (72.5%)	0.686	79 (69.9%)	157 (72.4%)	0.736
History of smoking	232 (70.3%)	85 (67.5%)	147 (72.1%)	0.445	80 (70.8%)	152 (70.0%)	0.988
History of HA exposure	37 (11.2%)	13 (10.3%)	24 (11.8%)	0.822	9 (8.0%)	28 (12.9%)	0.244
SpO_2_ at rest, %	98.00 [97.00, 99.00]	98.00 [97.00, 99.00]	98.00 [97.00, 99.00]	0.852	98.00 [97.00, 99.00]	98.00 [97.00, 99.00]	0.516
HR at rest, bpm	70.49 [66.77, 74.94]	70.27 [66.55, 74.23]	70.56 [67.05, 74.99]	0.636	71.00 [66.60, 74.40]	70.33 [66.91, 74.94]	0.941
SBP at rest, mmHg	116.95 [111.44, 121.58]	118.77 [111.75, 122.04]	116.28 [111.24, 121.22]	0.088	119.16 [111.95, 122.81]	116.29 [110.63, 121.06]	0.031
DBP at rest, mmHg	68.33 [65.06, 71.03]	68.40 [65.10, 71.28]	68.30 [65.06, 70.89]	0.992	68.53 [65.47, 71.29]	68.07 [64.93, 70.88]	0.475
SpO_2_ after exercise, %	95.00 [93.00, 96.00]	93.00 [92.00, 95.00]	95.00 [94.00, 97.00]	<0.001	93.00 [92.00, 95.00]	95.00 [94.00, 97.00]	<0.001
HR after exercise, bpm	90.00 [85.00, 97.75]	96.00 [87.00, 104.00]	88.50 [85.00, 93.00]	<0.001	97.00 [87.00, 104.00]	89.00 [85.00, 93.00]	<0.001
SBP after exercise, mmHg	121.00 [115.00, 129.00]	123.00 [114.00, 131.00]	120.00 [115.00, 127.00]	0.062	124.00 [115.00, 133.00]	120.00 [115.00, 127.00]	0.024
DBP after exercise, mmHg	74.00 [70.00, 77.00]	73.00 [70.25, 76.00]	74.00 [70.00, 77.00]	0.713	73.00 [71.00, 77.00]	74.00 [70.00, 77.00]	0.912
E/A	1.71 [1.52, 1.99]	1.74 [1.57, 1.93]	1.67 [1.49, 2.01]	0.433	1.75 [1.58, 1.95]	1.67 [1.48, 1.99]	0.196
E/e’	6.42 ± 0.99	6.37 ± 1.02	6.45 ± 0.97	0.491	6.35 ± 1.02	6.46 ± 0.98	0.345
EDV, mL	111.33 [98.03, 122.50]	110.46 [97.00, 121.62]	112.19 [99.57, 124.02]	0.316	110.09 [96.02, 119.99]	112.44 [99.59, 124.57]	0.101
ESV, mL	42.27 ± 7.90	41.92 ± 8.49	42.49 ± 7.52	0.244	41.47 ± 8.41	42.69 ± 7.61	0.063
EF, %	61.63 [60.00, 63.93]	61.64 [60.37, 63.92]	61.26 [59.52, 63.93]	0.205	62.06 [60.44, 63.93]	61.16 [59.53, 63.93]	0.101
CO, L/min	4.49 [4.01, 5.21]	4.50 [4.01, 5.18]	4.48 [4.01, 5.33]	0.900	4.37 [4.01, 5.16]	4.49 [4.02, 5.35]	0.684
GLS, %	20.47 ± 1.55	20.38 ± 1.53	20.53 ± 1.57	0.478	20.36 ± 1.56	20.53 ± 1.55	0.356
GCS, %	25.55 [24.23, 26.84]	25.44 [23.93, 26.79]	25.65 [24.56, 26.94]	0.074	25.48 [23.89, 26.84]	25.62 [24.42, 26.87]	0.244
Torsion, ^0^	10.93 [9.71, 12.28]	10.92 [9.74, 12.07]	10.93 [9.63, 12.38]	0.877	10.95 [9.85, 12.06]	10.91 [9.63, 12.38]	0.990
Untwisting rate, ^0^/s	−78.73 ± 21.95	−78.41 ± 22.32	−78.93 ± 21.77	0.687	−78.81 ± 22.98	−78.69 ± 21.45	0.820
QRS, ms	80.52 ± 10.66	81.32 ± 10.56	80.04 ± 10.69	0.177	81.55 ± 10.48	80.00 ± 10.71	0.166
QT, ms	384.06 ± 23.00	381.40 ± 24.53	385.70 ± 21.92	0.115	381.71 ± 25.40	385.28 ± 21.62	0.214
QT_c_, ms	404.28 ± 25.06	402.28 ± 27.37	405.51 ± 23.50	0.345	403.27 ± 27.92	404.80 ± 23.48	0.695
TpTe, ms	96.58 ± 9.39	96.05 ± 8.94	96.97 ± 9.65	0.358	95.76 ± 9.11	97.07 ± 9.51	0.178
TpTe_c_, ms	101.73 ± 10.24	101.34 ± 10.01	101.97 ± 10.40	0.739	101.20 ± 10.05	102.00 ± 10.35	0.667
TpTe/QT	0.25 ± 0.03	0.25 ± 0.03	0.25 ± 0.03	0.748	0.25 ± 0.03	0.25 ± 0.03	0.884
QT dispersion, ms	32.48 ± 8.94	33.41 ± 8.39	31.90 ± 9.24	0.112	33.82 ± 8.04	31.78 ± 9.32	0.036
Corrected QT dispersion, ms	34.22 ± 9.58	35.30 ± 9.13	33.55 ± 9.82	0.096	35.79 ± 8.78	33.40 ± 9.90	0.025
TpTe dispersion, ms	24.43 ± 8.26	24.04 ± 8.66	24.67 ± 8.01	0.379	23.81 ± 8.67	24.76 ± 8.03	0.214
Corrected TpTe dispersion, ms	25.72 ± 8.75	25.33 ± 9.07	25.97 ± 8.57	0.457	25.11 ± 9.06	26.04 ± 8.60	0.307
AT-RCP time, s	170.21 [158.10, 182.06]	157.10 [147.41, 168.06]	175.91 [168.12, 186.70]	<0.001	156.16 [146.67, 166.04]	175.08 [167.63, 186.10]	<0.001
Peak VO_2_,mL/min/kg	32.99 ± 2.95	31.27 ± 2.48	34.06 ± 2.70	<0.001	31.26 ± 2.53	33.89 ± 2.75	<0.001
VO_2_ at AT, mL/min/kg	14.98 ± 1.40	15.56 ± 1.25	14.62 ± 1.37	<0.001	15.59 ± 1.20	14.66 ± 1.39	<0.001
VO_2_ at RCP, mL/min/kg	26.46 [25.29, 27.62]	25.42 [24.18, 26.55]	26.90 [25.92, 28.41]	<0.001	25.26 [24.10, 26.45]	26.88 [25.88, 28.32]	<0.001
VO_2_/WR slope, mL/min/W	9.39 [8.77, 9.98]	9.82 [9.16, 10.27]	9.20 [8.50, 9.72]	<0.001	9.73 ± 0.78	9.15 ± 0.93	<0.001
ΔVO_2_ AT-RCP, mL/min	11.39 [9.93, 13.19]	9.90 [8.45, 11.21]	12.38 [11.09, 13.91]	<0.001	9.57 [8.37, 10.88]	12.27 [11.07, 13.80]	<0.001

Data were expressed as *n* (%), median [IQR] and mean ± SD; *p* value, AMS vs. Non-AMS. AMS, acute mountain sickness; AT, anaerobic threshold; BMI, body mass index; CO, cardiac output; DBP, diastolic blood pressure; EDV, end-diastolic volume; EF, ejection fraction; ESV, end-systolic volume; E/A, peak early diastolic velocity/late diastolic velocity; E/e’, peak early diastolic velocity/early diastolic velocity; GCS, global circumferential strain; GLS, global longitudinal strain; HA, high altitude; HR, heart rate; IQR, interquartile range; LLS, Lake Louise Score; QRS, QRS wave width; QT, QT interval; QT_c_, corrected QT interval; RCP, respiratory compensation point; SBP, systolic blood pressure; SD, standard deviation; SpO_2_, saturation of peripheral oxygen; TpTe. T wave peak to end interval; TpTe_c_, corrected T wave peak to end interval; WR, work rate.

### Model development and selection

Five ML models were trained and evaluated to predict 4 clinical outcomes. For the prediction of AMS diagnosed by the old LLS, the RF model achieved the best performance [AUC = 0.952, 95% confidence interval (CI): 0.875–0.998]; For the prediction of AMS diagnosed by the new LLS, RF model again performed best (AUC = 0.959, 95% CI: 0.910–0.992); For the prediction of AMS score using the old LLS, AdaBoost (AUC = 0.962, 95% CI: 0.902–1.000) and RF models (AUC = 0.962, 95% CI: 0.904–0.998) yielded top-performing; For the prediction of AMS score using the new LLS, the LightGBM model showed the best performance (AUC = 0.950, 95% CI: 0.900–0.990) (Figure 1). Comprehensive comparisons of all 5 models were also presented. Regarding the prediction of AMS occurrence, we evaluated the 5 models based on the calculated parameters of accuracy rate, precision rate, recall rate, and F1 score (Supplementary Table 1). Regarding the prediction of AMS score, we evaluated the 5 models based on the calculated parameters of MAE, RMSE, and *R*^2^ (Supplementary Table 2). Based on the comparative results of 5 ML models, we have decisively selected the RF, AdaBoost, and LightGBM models as our optimal choices and employed them to construct a AMS-FTE model.

**Figure 1:**
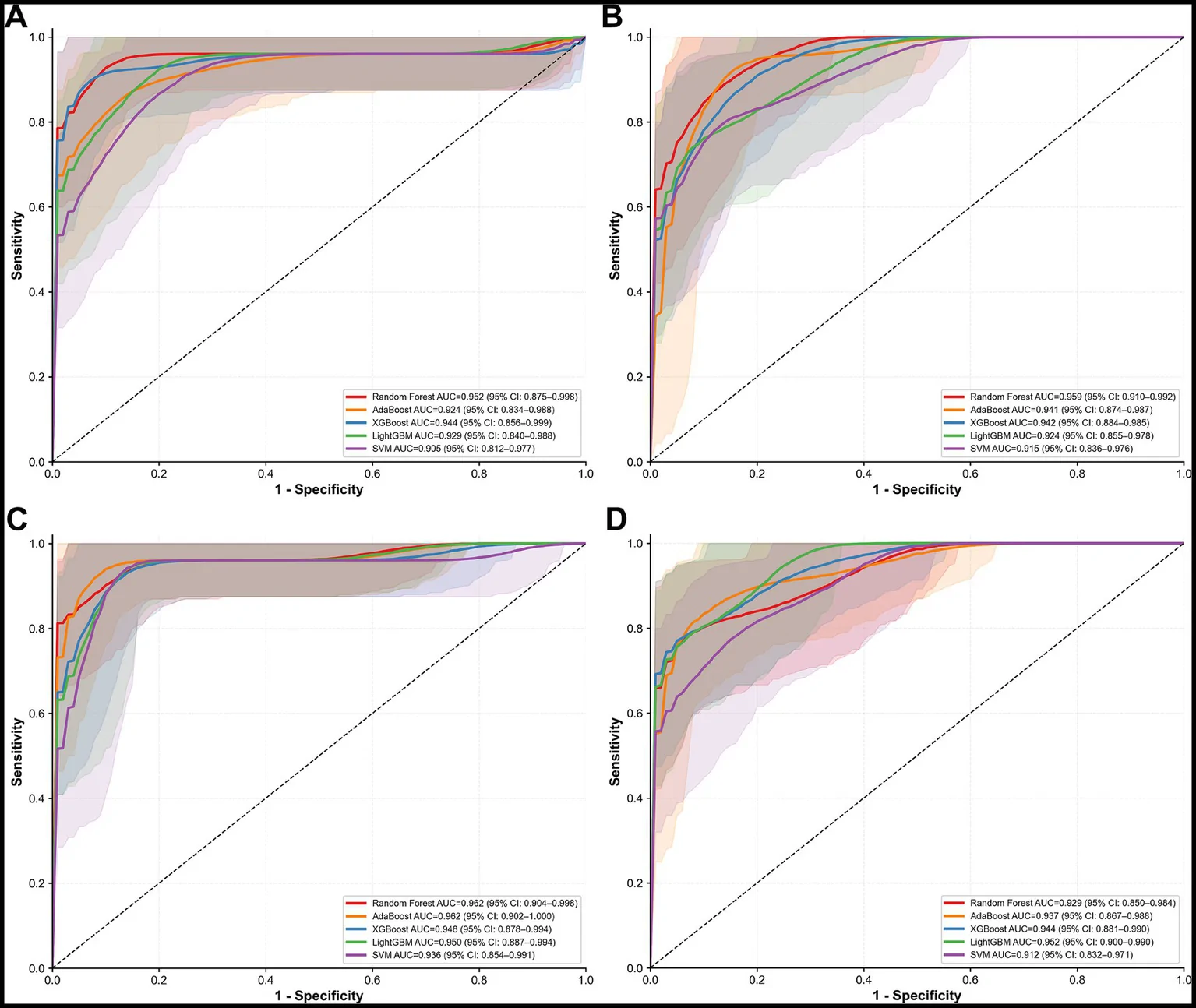
Performance of ML models for prediction of clinical outcomes. ROC curves comparing the performance of 5 ML models across 4 prediction tasks: **(A)** AMS diagnosed by the old LLS, **(B)** AMS diagnosed by the new LLS, **(C)** AMS score using the old LLS and **(D)** AMS score using the new LLS. AdaBoost, adaptive boosting; AMS, acute mountain sickness; AUC, area under the ROC curve; LightGBM, light gradient boosting machine; LLS, Lake Louise Score; ML, machine learning; RF, random forest; ROC, receiver operating characteristic; SVM, support vector machine; XGBoost, extreme gradient boosting.

### Feature selection based on SHAP

SHAP analysis was performed to quantify the contribution of each input variable for the task-specific AMS-FTE model predictions. Global feature importance was determined using the mean absolute SHA*p* value within each task, while the SHAP summary plots illustrated both the magnitude and direction of each variable’s effect on the model output (Figure 2). When the results of the four tasks were considered collectively, 5 persistently dominant features: HR after exercise, SpO_2_ after exercise, AT-RCP time, peak VO_2_, and ΔVO_2_ AT-RCP, which were associated with cardiorespiratory fitness showed the most consistent overall contributions to the ensemble predictions (Figure 3). Furthermore, to determine an appropriate feature-set size, AMS-FTE model constructed using the top 3, 5, 7, 9, 11, and 13 SHAP-ranked variables were compared across the four prediction tasks (Supplementary Figure 1). The five-feature configuration provided the most consistent overall discrimination: it achieved the highest AUCs for the two old-LLS tasks and showed performance close to the task-specific maxima for the two new-LLS tasks. Notably, the small changes in AUC after feature reduction suggest that the five selected features retained most of the discriminative information, whereas lower-ranked variables provided relatively limited complementary contribution. Therefore, the final five-feature subset was selected by balancing predictive discrimination, model parsimony, clinical interpretability, and measurement burden. Based on the subset, the AMS-FTE model achieved AUCs of 0.947 (95% CI: 0.862–0.997) for old-LLS AMS classification, 0.948 (95% CI: 0.896–0.988) for new-LLS AMS classification, 0.963 (95% CI: 0.905–0.999) for old-LLS score prediction, and 0.945 (95% CI: 0.879–0.991) for new-LLS score prediction. These findings indicate that the compact five-feature models retained high discriminative performance while improving interpretability and reducing the number of required measurements (Figure 4).

**Figure 2:**
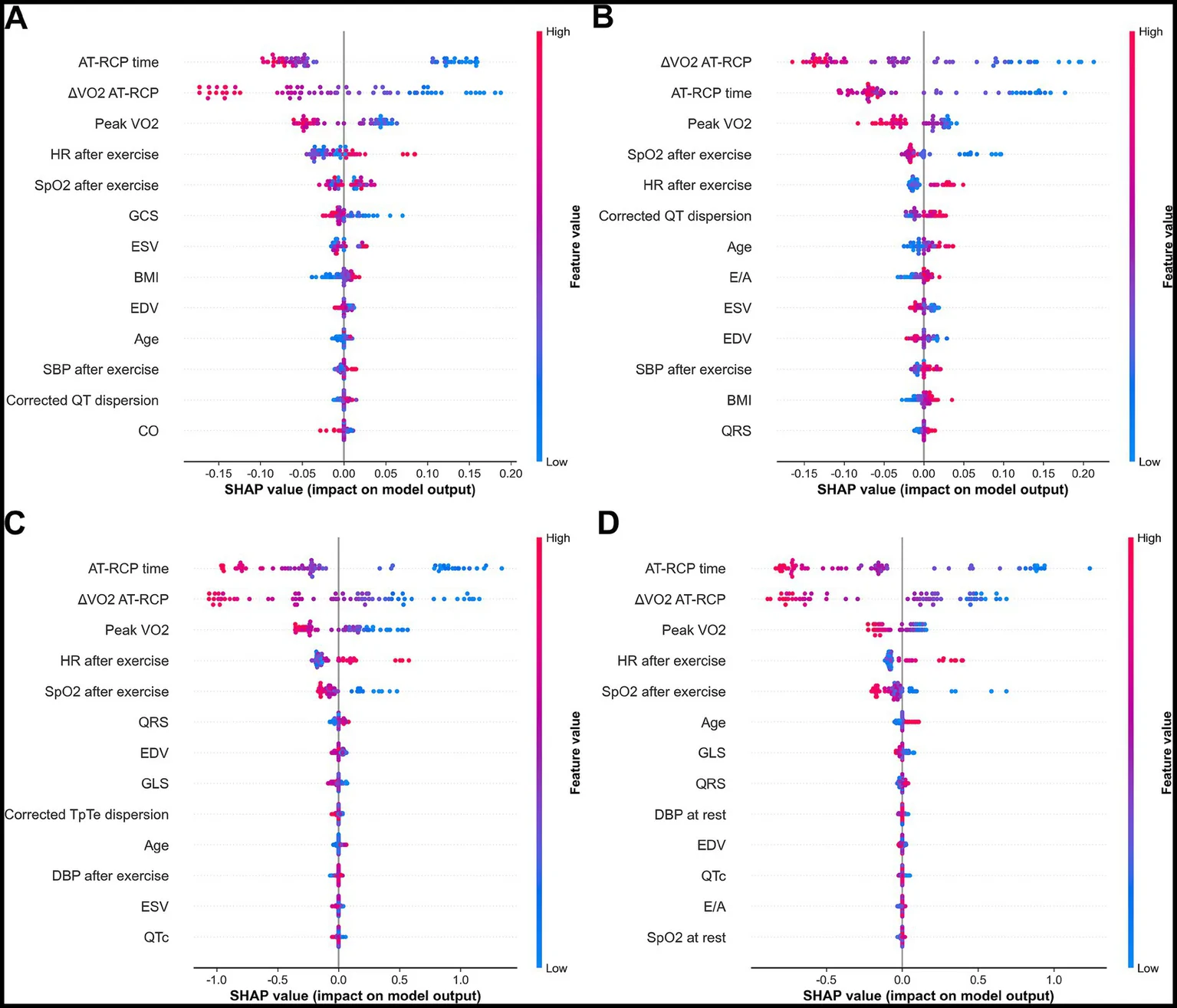
Global interpretability of the AMS-FTE model using SHAP. SHAP summary plots for the AMS-FTE model across 4 prediction tasks: **(A)** AMS diagnosed by the old LLS, **(B)** AMS diagnosed by the new LLS, **(C)** AMS score using the old LLS, and **(D)** AMS score using the new LLS. AMS, Acute mountain sickness; AMS-FTE, Acute Mountain Sickness Four-Task Ensemble; LLS, Lake Louise Score; SHAP, SHapley Additive exPlanations.

**Figure 3:**
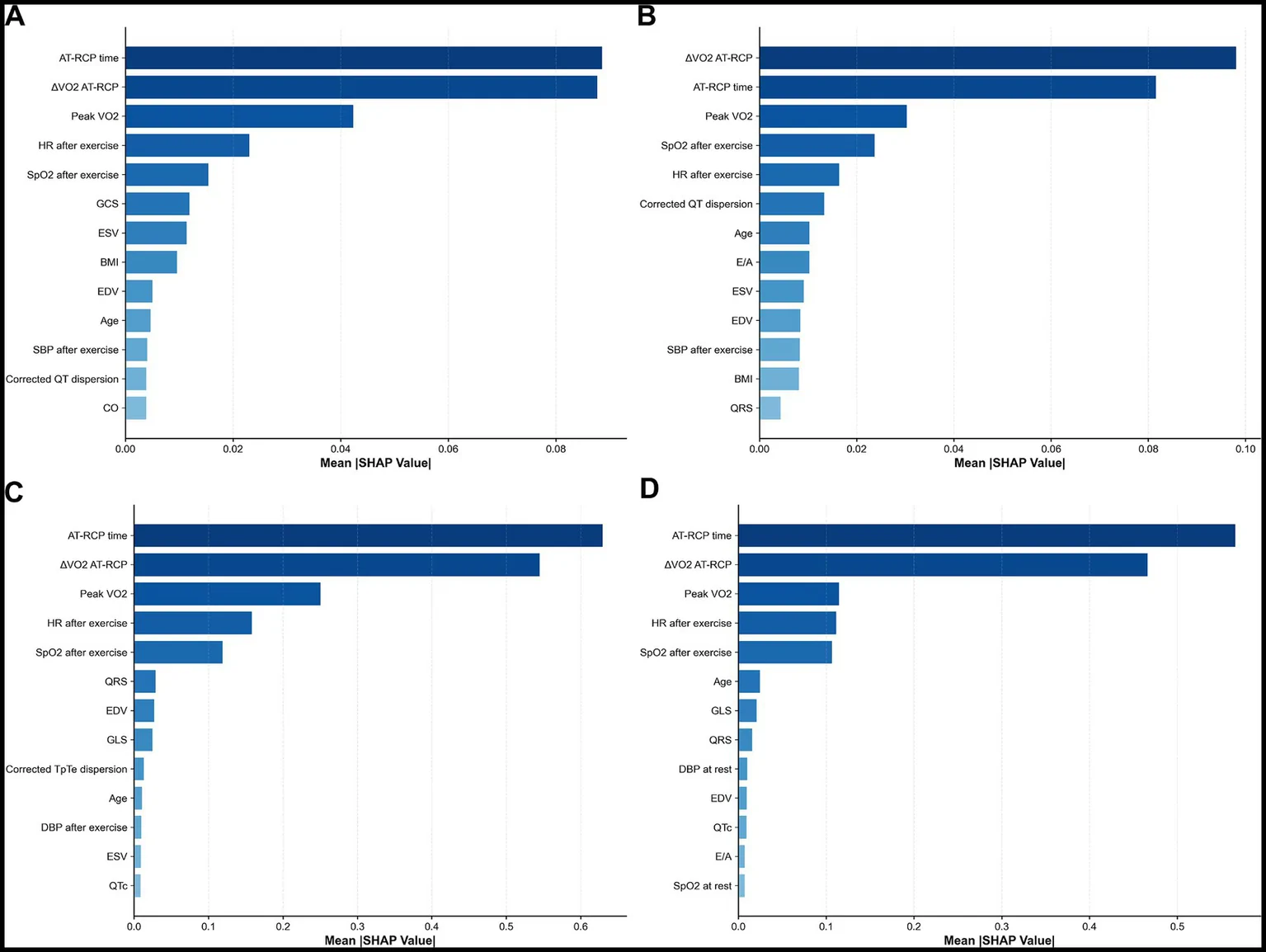
Mean absolute SHA*p* value of the AMS-FTE model. Mean absolute SHAP value for the AMS-FTE model across 4 prediction tasks: **(A)** AMS diagnosed by the old LLS, **(B)** AMS diagnosed by the new LLS, **(C)** AMS score using the old LLS, and **(D)** AMS score using the new LLS. AMS, Acute mountain sickness; AMS-FTE, Acute Mountain Sickness Four-Task Ensemble; LLS, Lake Louise Score; SHAP, SHapley Additive exPlanations.

**Figure 4:**
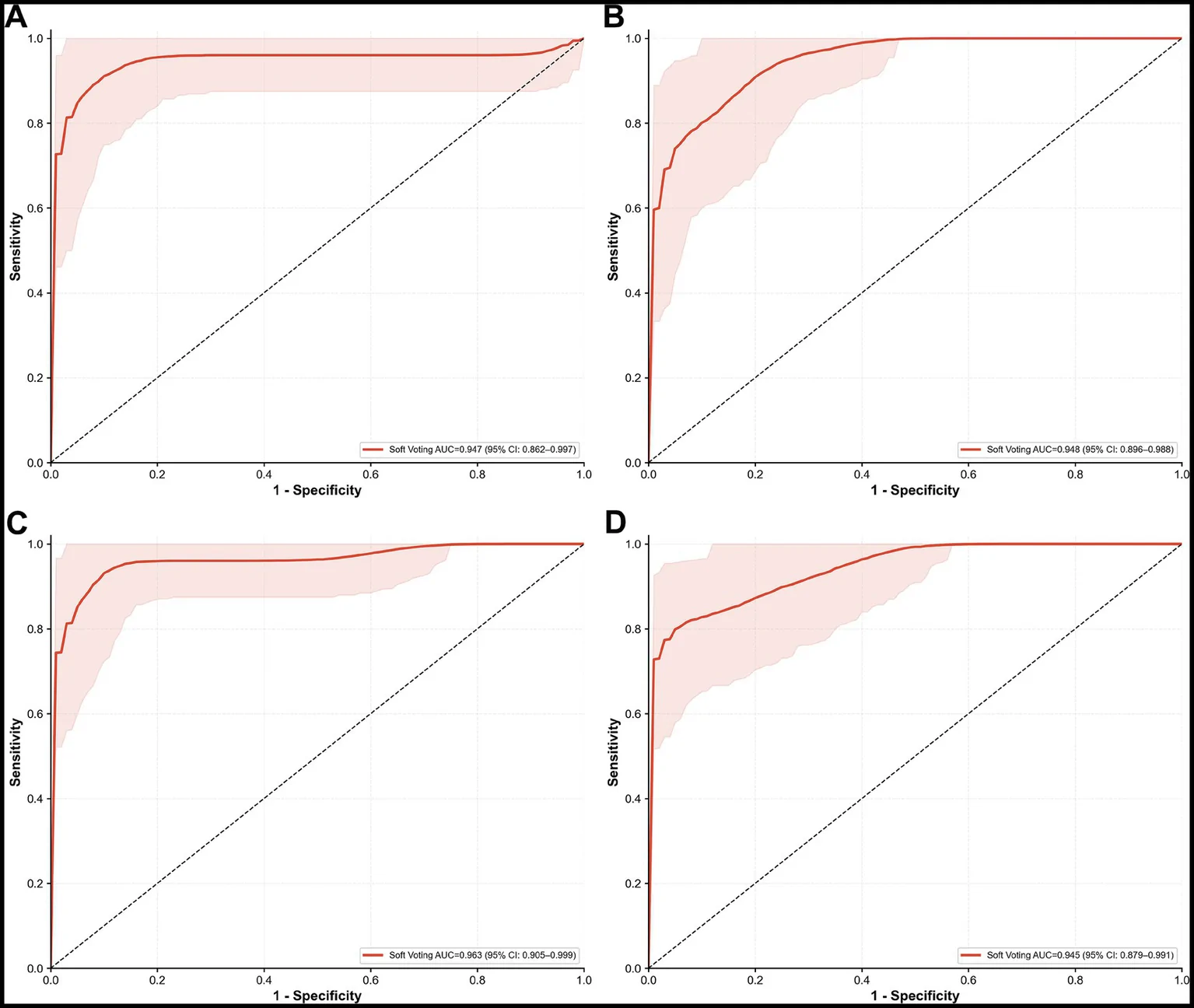
AUCs for the AMS-FTE model across five selected features in each prediction task. ROC curves of 5 selected features in the AMS-FTE model across 4 prediction tasks: **(A)** AMS diagnosed by the old LLS, **(B)** AMS diagnosed by the new LLS, **(C)** AMS score using the old LLS and **(D)** AMS score using the new LLS. AMS, acute mountain sickness; AMS-FTE, Acute Mountain Sickness Four-Task Ensemble; AUC, rea under the ROC curve; LLS, Lake Louise Score; ROC, eceiver operating characteristic.

### Evaluation of selected key features

To further evaluate the clinical utility of the final five-feature AMS-FTE model, DCA was performed using fivefold out-of-fold predictions. DCA calculates the clinical NB across a full spectrum of threshold probabilities by balancing true-positive against false-positive outcomes. Results demonstrated that under the old LLS evaluation systems, the AMS-FTE model achieved a clinically significant NB surpassing zero across the probability ranges of 0.041 to 0.922 and 0.047 to 0.937. Under the new LLS evaluation systems, the model also yielded a substantial NB exceeding 0 across the probability ranges of 0.005 to 0.807 and 0.007 to 0.790. These findings robustly confirm the model’s strong clinical utility across both systems. Incremental NB values at threshold probabilities of 10, 20, and 30% are reported to facilitate clinical interpretation: for predicting AMS diagnosed by the old LLS (0.011, 0.066, 0.152); for predicting AMS diagnosed by the new LLS (0.013, 0.082, 0.167); for predicting AMS severity assessed by the old LLS (0.017, 0.058, 0.145); for predicting AMS severity assessed by the new LLS (0.019, 0.068, 0.184) (Figure 5). Moreover, to promote clinical implementation, we developed a support system powered using the final five-feature AMS-FTE model, available in a web-based format. As Figure 6 illustrated, this system could predict the onset and severity of AMS based on both the old and new versions of the LLS.

**Figure 5:**
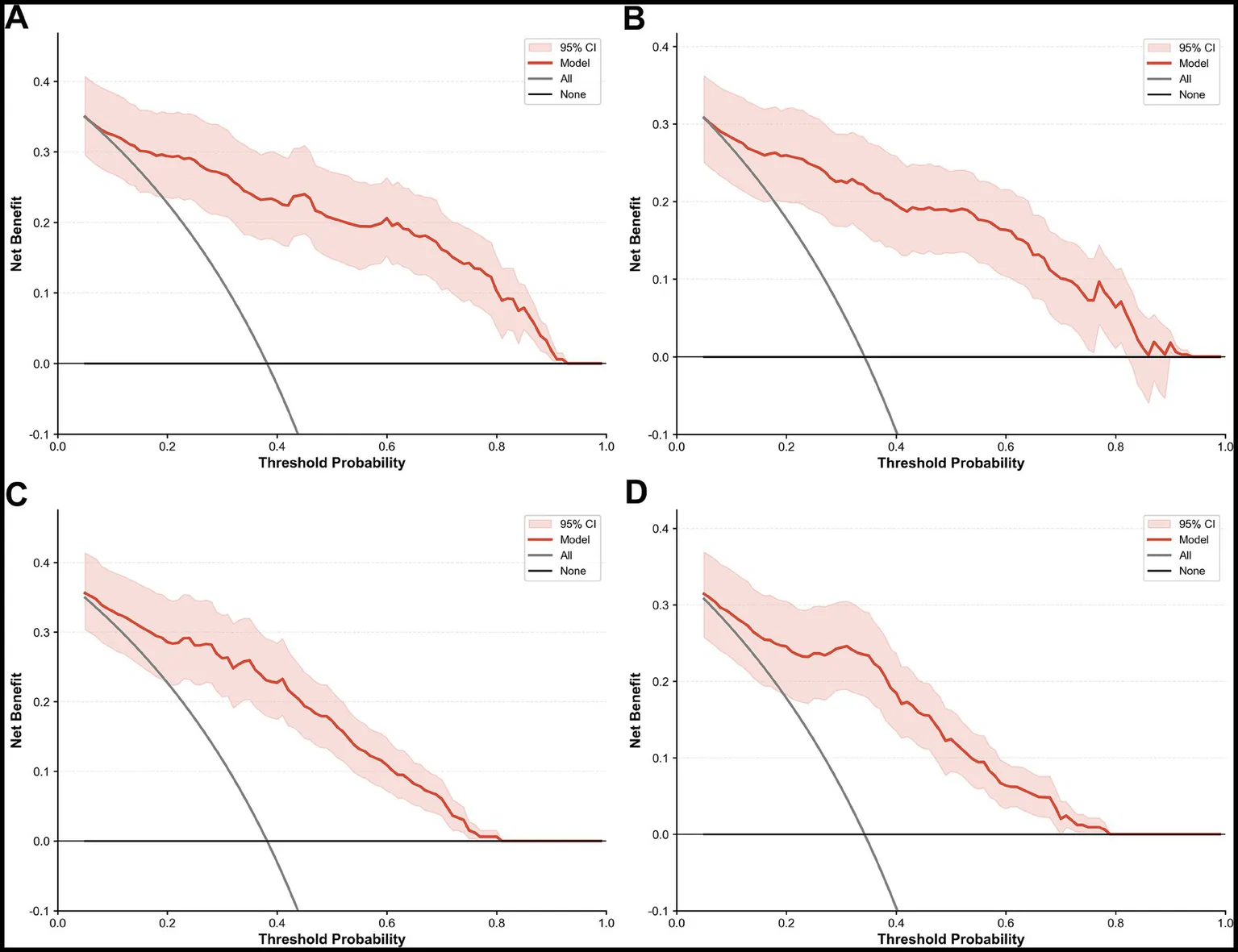
DCA for the AMS-FTE model across five selected features in each prediction task. DCA of 5 selected features in the AMS-FTE model across 4 prediction tasks: **(A)** AMS diagnosed by the old LLS, **(B)** AMS diagnosed by the new LLS, **(C)** AMS score using the old LLS and **(D)** AMS score using the new LLS. AMS, Acute mountain sickness. AMS-FTE, Acute mountain sickness four-task ensemble. DCA, Decision curve analysis. LLS, Lake Louise score.

**Figure 6:**
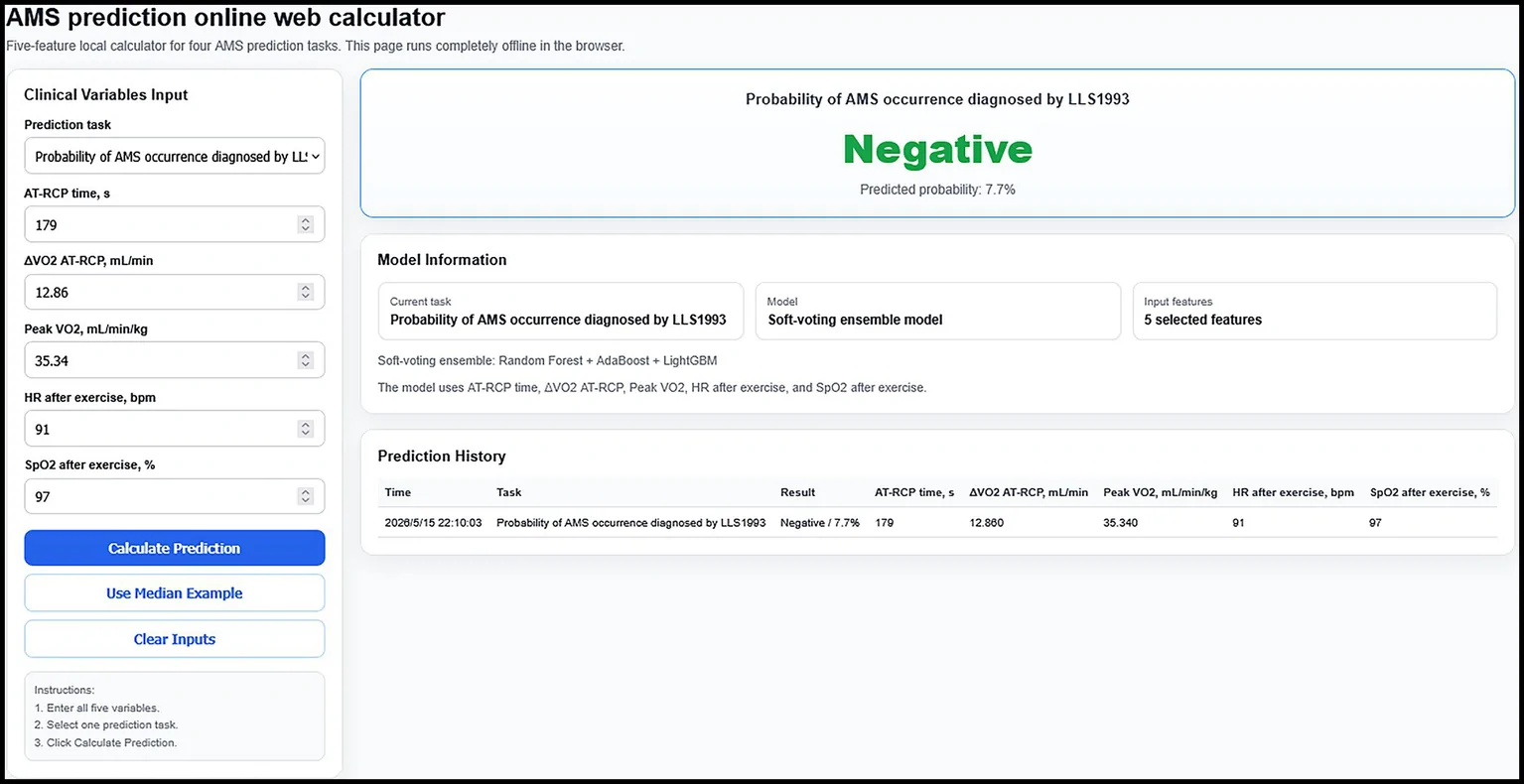
Overview of the SHAP-based AMS prediction support system. User interfaces of the AMS prediction support system designed to support clinical implementation. This system employs a AMS-FTE model, integrating 5 variables including post-exercise HR, post-exercise SpO_2_, AT-RCP time, peak VO_2_, and ΔVO_2_ AT-RCP to predict the incidence and severity of AMS. AMS, acute mountain sickness; AMS-FTE, acute mountain sickness four-task ensemble; AT, anaerobic threshold; HR, heart rate; RCP, respiratory compensation point; SHAP, SHapley additive explanations; SpO_2_, percutaneous oxygen saturation.

## Discussion

With the surging popularity of outdoor pursuits like hiking, skiing, and mountaineering, the impact of AMS has become increasingly widespread (22). This condition not only jeopardizes individual health but also carries grave consequences for community well-being, economic stability, and medical resource allocation. AMS describes an array of intricate physiological responses triggered when the body rapidly ascends to high altitudes, confronting diminished oxygen levels and shifting atmospheric pressure (23–26). Individual susceptibility to AMS differs markedly, and severe cases can prove potentially fatal, rendering the early identification critically important (22). Hypoxemia, resulting from reduced oxygen partial pressure at higher altitudes, triggers insufficient oxygen supply to tissues (27). This consequently initiates cerebral vasodilation, elevates intracranial pressure, and produces headaches-the hallmark symptom of AMS (22, 28). Furthermore, hypobaric hypoxia conditions induce pulmonary vasoconstriction, increasing pulmonary artery pressure and exacerbating symptoms like coughing and dyspnea (29). When airflow and blood flow within the lungs become mismatched, gas exchange is significantly impaired. This impairment compels cells to switch from aerobic to anaerobic metabolism, generating lactic acid that causes pronounced fatigue and muscle soreness (30). Thus, a weakened hypobaric hypoxia response or reduced oxygen delivery efficiency lies at the heart of AMS development, where optimal cardiopulmonary function undoubtedly plays a pivotal protective role. For instance, individuals with impaired pulmonary function or mild respiratory diseases often find it particularly challenging to adapt to hypoxic environments, significantly elevating their AMS risk (31). Furthermore, cardiovascular dysfunction can profoundly disrupt oxygen delivery to vital tissues, intensifying the body’s reaction to HA hypoxia and markedly worsening typical AMS symptoms (32).

Cardiopulmonary fitness signifies the cardiovascular and respiratory systems’ capacity to deliver oxygen to tissues while preventing metabolic waste accumulation, serving as a key indicator of the body’s overall efficiency in oxygen utilization (33). Researches have consistently shown that cardiopulmonary fitness is strongly linked to the risk of developing numerous diseases and all-cause mortality, with its predictive power often surpassing many traditional risk factors (34–39). Evaluating cardiopulmonary fitness in susceptible populations is therefore essential for preventing, detecting early, or diagnosing potential health issues. The CPET remains the definitive gold standard for this assessment, comprehensively gaging heart and lung function through precise measurements of VO_2_, AT, SpO_2_ and so on (10, 40). Recent studies further indicate these CPET-derived metrics can effectively evaluate an individual’s tolerance to HA hypobaric hypoxia environments, potentially reducing the risk of AMS (9, 41). Peak VO_2_ signifies an individual’s maximum oxygen utilization capacity. Under normoxic conditions, a higher peak VO_2_ reflects greater physical exercise capacity and enhanced cardiorespiratory fitness. Consequently, baseline peak VO_2_ serves as a robust predictor for AMS development and effectively identifies susceptible individuals following rapid exposure to HA (41). This conclusion aligns with our study’s findings. AT-RCP time represents the capacity to regulate acid–base balance, known as isocapnic buffering capacity (IBC). During this stage, lactate surges within the muscles, though it is quickly neutralized by bicarbonate. Consequently, a longer AT-RCP time reduces the risk of metabolic acidosis, enabling athletes to sustain high-intensity exercise longer. Conversely, a short AT-RCP phase or a premature onset of RCP (insufficient IBC) often correlates with breathlessness and premature fatigue, which may potentially intensifying AMS symptoms (13, 42). While our data revealed a robust statistical association between IBC (represented by AT-RCP time and ΔVO_2_ AT-RCP) and AMS incidence, the direct pathophysiological pathway warrants careful interpretation. We hypothesize that a diminished IBC reflects a systemic vulnerability to metabolic acidosis. Under hypobaric hypoxia, individuals with limited buffering reserves may experience accelerated lactate accumulation, potentially exacerbating the cerebral acidosis implicated in AMS pathogenesis. However, as this was an observational clinical study, these parameters should be regarded as functional surrogates for metabolic resilience rather than direct causative agents. Future mechanistic studies involving serial blood lactate measurements or cerebrospinal fluid analysis are required to substantiate this link. Post-exercise physiological indicators offer an invaluable window into cardiopulmonary fitness, and prior research has established their utility in predicting AMS (41, 43). Our findings showed that post-exercise HR and SpO_2_ serve as robust predictors of AMS. These metrics point to the recovery kinetics of the autonomic nervous system and the efficiency of gas exchange—key components of cardiopulmonary reserve. Resting measurements reflect a state of minimal metabolic demand where homeostatic mechanisms can readily mask subclinical inefficiencies. In contrast, CPET imposes a significant hemodynamic and ventilatory stress, functioning as a physiological stress test. The acute decompensation observed post-exercise (manifested by a sharper decline in SpO_2_ and attenuated HR recovery in the AMS group) suggests a latent inability to dynamically augment cardiac output and ventilatory drive to meet heightened oxygen demands. This failure to maintain homeostasis under stress parallels the physiological challenge of acute hypobaric hypoxia exposure. Consequently, cardiopulmonary reserve capacity derived from dynamic indices appear to be more sensitive barometers of altitude resilience than static resting parameters, as they unmask maladaptive responses that remain dormant at baseline. However, as these values are sensitive to immediate physiological states, the additional predictive power of post-exercise responses over static baseline values merits further investigation in future studies.

ML offers a powerful computational approach and has demonstrated significant efficacy in discovering the most predictive risk factors, thus researchers can develop robust risk models (16, 44). The SHAP method enhances interpretability by offering comprehensive insights on feature significance and the roles of specific variables, rendering it a more efficacious instrument for clinical applications (19, 20). Despite the current scarcity of research, ML models are rapidly gaining traction in AMS prediction. Wei et al. ingeniously employ ML algorithms to meticulously track real-time physiological and environmental data from subjects in HA conditions. Their work effectively demonstrates the potential for accurate AMS prediction (45). Gesang et al. achieved accurate prediction of the severity of HAPE in patients through model comparison, using a RF model combined with 4 key indicators: lung rales, sputum production, HR, and SpO_2_ (46). The two previous studies have successfully deployed ML models utilizing real-time environmental and clinical parameters to monitor physiological status or detect severe HAPE post-exposure, reporting AUCs ranging from 0.85 to 0.92. While these models offer utility in acute care settings, their reliance on data collected after ascent limits their utility for preemptive intervention. Conversely, Yang et al. devised a genetic screening tool for severe AMS, using microarray data from peripheral blood mononuclear cell samples of 21 subjects and harnessing a support vector machine recursive feature elimination (SVM-RFE) system for ML, and 10 feature genes exhibiting robust predictive capacity (47); Li et al. leveraged the integration of clinical, proteomic, and metabolomic data, applying the mutual information-radial SVM-RFE method to identify key biomarkers. Specifically selected were 2 clinical features (e.g., peak expiratory flow), six proteins (e.g., ACSL4), and 4 metabolites (e.g., 2-methyl-1,3-cyclohexadiene). They developed an AMS prediction model and rigorously evaluated its performance using an independent validation cohort, which demonstrated robust predictive capability (AUC: 0.94). This approach paves the way for shifting from passive healthcare toward a predictive, preventive, and personalized medical paradigm, establishing a foundation for pre-exposure risk screening and intervention strategies in HA environments (48). However, the logistical complexity and economic burden of omics technologies may impede their widespread clinical adoption for routine screening. In contrast, our study leveraged standardized CPET metrics, which readily obtainable in clinical practice, to achieve comparable or superior discriminatory power (AUC: 0.947–0.963). Crucially, our AMS-FTE framework uniquely addresses both disease incidence and severity grading using two internationally validated scoring systems (the 1993 and 2018 versions of the LLS), thereby bolstering the credibility of our findings. By identifying high-risk individuals based on baseline physiological reserve prior to altitude exposure, our approach facilitates a shift from reactive management to proactive, personalized prevention strategies.

### Limitations

Nevertheless, this study has several limitations. First, post-exercise indicators may be affected by random factors, leading to unstable measurement values, thus future studies should incorporate resting baseline values to isolate the additive predictive value of post-exercise responses. Second, while this study identified significant predictive associations, it cannot establish direct causality between CPET indices and AMS onset. The proposed links between IBC and AMS pathophysiology remain theoretical. The absence of concurrent arterial blood gas analysis or lactate measurements during CPET limits our ability to elucidate the precise biochemical mechanisms by which variations in IBC translate into clinical AMS symptoms. Basic mechanistic experiments are necessary to validate these hypothesized pathways. Third, the cohort consisted exclusively of young men from China and was predominantly Han Chinese, the analysis of other age groups and races remains insufficient. And the relatively small sample size also constrains the wider generalizability of these findings. Finally, although we employed rigorous internal cross-validation and held out a separate testing set, the absence of an independent external validation cohort remains a important limitation. These outputs from the algorithms should be interpreted as model-derived risk estimates rather than externally calibrated absolute probabilities. Consequently, future large-scale, multi-center prospective studies are strongly recommended.

## Conclusion

In conclusion, we introduced a robust AMS-FTE model designed to predict both the onset and severity of AMS. This AMS-FTE model emerged from systematically comparing optimal ML algorithms enhanced with SHAP techniques to guide feature selection, dramatically enhancing its interpretability and clinical utility. The core predictive indicators identified include HR after exercise, SpO_2_ after exercise, AT-RCP time, peak VO_2_, and ΔVO_2_ AT-RCP. Crucially, our study revealed that baseline cardiorespiratory fitness indices offer strong predictive value for AMS risk. This enables earlier diagnosis and personalized interventions for altitude-related disorders, thereby optimizing the allocation of critical medical resources.

## Data Availability

The raw data supporting the conclusions of this article will be made available by the authors, without undue reservation.

## Glossary

A: Late diastolic velocity
ABP: Ambulatory blood pressure
AdaBoost: Adaptive boosting
AMS: Acute mountain sickness
AMS-FTE: Acute Mountain Sickness Four-Task Ensemble
AT: Anaerobic threshold
AUC: Area under the receiver operating characteristic curve
BMI: Body mass index
BP: Blood pressure
CI: Confidence interval
CPET: Cardiopulmonary exercise testing
DCA: Decision curve analysis
EF: Ejection fraction
E: Peak early diastolic velocity
e’: Early diastolic velocity
ECG: Electrocardiogram
GLS: Global longitudinal strain
GCS: Global circumferential strain
HA: High altitude
HACE: High altitude cerebral edema
HAPE: High altitude pulmonary edema
HR: Heart rate
IBC: Isocapnic buffering capacity
IQR: Interquartile range
LA: Low altitude
LightGBM: Light gradient boosting machine
LLS: Lake Louise Score
MAE: Mean absolute error
ML: Machine learning
NB: Net benefit
QTc: Corrected QT interval
RCP: Respiratory compensation point
RF: Random forest
RMSE: Root mean square error
SBP: Systolic blood pressure
SD: Standard deviation
SHAP: SHapley Additive exPlanations
SpO_2_: Percutaneous oxygen saturation
SVM: Support vector machine
SVM-RFE: Support vector machine recursive feature elimination
TpTec: Corrected TpTe interval
WR: Work rate
XGBoost: Extreme gradient boosting
