# Supplementary material for: Baseline indices of cardiorespiratory fitness for predicting acute mountain sickness: an interpretable four-task ensemble model
Source: Front Public Health. 2026 Sep 22;14:1883321. doi: 10.3389/fpubh.2026.1883321 (PMC13638543; doi:10.3389/fpubh.2026.1883321)
Supplement: Supplementary file 1 [file Table_1.DOCX]

**Supplemental table 1. Comparison of diagnostic performance of AMS occurrence.**

| **Model** | **LLS** | **Accuracy (%)** | **Precision (%)** | **Recall (%)** | **F1-Score (%)** |
| --- | --- | --- | --- | --- | --- |
| XGBoost | Old | 86.36 | 86.36 | 76.00 | 80.85 |
|  | New | 84.85 | 88.24 | 65.22 | 75.00 |
| AdaBoost | Old | 93.94 | 92.00 | 92.00 | 92.00 |
|  | New | 84.85 | 84.21 | 69.57 | 76.19 |
| RF | Old | 89.39 | 100.00 | 72.00 | 83.72 |
|  | New | 89.39 | 86.36 | 82.61 | 84.44 |
| LightGBM | Old | 87.88 | 90.48 | 76.00 | 82.61 |
|  | New | 84.85 | 88.24 | 65.22 | 75.00 |
| SVM | Old | 81.82 | 84.21 | 64.00 | 72.73 |
|  | New | 81.82 | 86.67 | 56.52 | 68.42 |

AdaBoost: adaptive boosting; AMS: acute mountain sickness; LightGBM: light gradient boosting machine; LLS: Lake Louise Score; RF: random forest; SVM: support vector machine; XGBoost: extreme gradient boosting.
