# Supplementary material for: Baseline indices of cardiorespiratory fitness for predicting acute mountain sickness: an interpretable four-task ensemble model
Source: Front Public Health. 2026 Sep 22;14:1883321. doi: 10.3389/fpubh.2026.1883321 (PMC13638543; doi:10.3389/fpubh.2026.1883321)
Supplement: Supplementary file 2 [file Table_2.DOCX]

**Supplemental table 2. Comparison of diagnostic performance of AMS severity.**

| **Model** | **LLS** | **RMSE** | **MAE** | **R²** |
| --- | --- | --- | --- | --- |
| XGBoost | Old | 1.097 | 0.872 | 0.678 |
|  | New | 1.072 | 0.804 | 0.659 |
| AdaBoost | Old | 1.071 | 0.832 | 0.693 |
|  | New | 0.970 | 0.697 | 0.720 |
| RF | Old | 1.098 | 0.868 | 0.677 |
|  | New | 0.972 | 0.713 | 0.719 |
| LightGBM | Old | 1.094 | 0.838 | 0.679 |
|  | New | 0.972 | 0.705 | 0.719 |
| SVM | Old | 1.407 | 1.124 | 0.502 |
|  | New | 1.238 | 0.961 | 0.590 |

AdaBoost: adaptive boosting; AMS: acute mountain sickness; LightGBM: light gradient boosting machine; LLS: Lake Louise Score; MAE: mean absolute error, RF: random forest; RMSE: root mean square error; SVM: support vector machine; XGBoost: extreme gradient boosting.
