# Supplementary material for: Baseline indices of cardiorespiratory fitness for predicting acute mountain sickness: an interpretable four-task ensemble model
Source: Front Public Health. 2026 Sep 22;14:1883321. doi: 10.3389/fpubh.2026.1883321 (PMC13638543; doi:10.3389/fpubh.2026.1883321)
Supplement: Supplementary file 3 [file Data_Sheet_1.ZIP › AMS prediction online web calculator.html]

AMS prediction online web calculator


# AMS prediction online web calculator

Five-feature local calculator for four AMS prediction tasks. This page runs completely offline in the browser.

Clinical Variables Input

Prediction task

Probability of AMS occurrence diagnosed by LLS1993
Probability of AMS occurrence diagnosed by LLS2018
Probability indicates a potentially severe AMS diagnosed by LLS1993
Probability indicates a potentially severe AMS diagnosed by LLS2018
AT-RCP time, s

ΔVO2 AT-RCP, mL/min

Peak VO2, mL/min/kg

HR after exercise, bpm

SpO2 after exercise, %

Calculate Prediction
Use Median Example
Clear Inputs

Instructions:  
1. Enter all five variables.  
2. Select one prediction task.  
3. Click Calculate Prediction.

Prediction Result

--

Enter the five features and calculate.

Model Information

Current task

Probability of AMS occurrence diagnosed by LLS1993

Model

Soft-voting ensemble model

Input features

5 selected features

Soft-voting ensemble model

The prediction is generated from a locally embedded Soft-voting ensemble model exported from the uploaded trained model.

Prediction History

| Time | Task | Result | AT-RCP time, s | ΔVO2 AT-RCP, mL/min | Peak VO2, mL/min/kg | HR after exercise, bpm | SpO2 after exercise, % |
| --- | --- | --- | --- | --- | --- | --- | --- |
